# Increased Gut Permeability and Microbiota Change Associate with Mesenteric Fat Inflammation and Metabolic Dysfunction in Diet-Induced Obese Mice

**DOI:** 10.1371/journal.pone.0034233

**Published:** 2012-03-23

**Authors:** Yan Y. Lam, Connie W. Y. Ha, Craig R. Campbell, Andrew J. Mitchell, Anuwat Dinudom, Jan Oscarsson, David I. Cook, Nicholas H. Hunt, Ian D. Caterson, Andrew J. Holmes, Len H. Storlien

**Affiliations:** 1 Boden Institute of Obesity, Nutrition, Exercise and Eating Disorders, University of Sydney, Sydney, Australia; 2 School of Molecular Bioscience, University of Sydney, Sydney, Australia; 3 Discipline of Physiology, Bosch Institute, University of Sydney, Sydney, Australia; 4 Molecular Immunopathology Unit, Bosch Institute and Sydney Medical School, University of Sydney, Sydney, Australia; 5 AstraZeneca R&D, Mölndal, Sweden; Wayne State University, United States of America

## Abstract

We investigated the relationship between gut health, visceral fat dysfunction and metabolic disorders in diet-induced obesity. C57BL/6J mice were fed control or high saturated fat diet (HFD). Circulating glucose, insulin and inflammatory markers were measured. Proximal colon barrier function was assessed by measuring transepithelial resistance and mRNA expression of tight-junction proteins. Gut microbiota profile was determined by 16S rDNA pyrosequencing. Tumor necrosis factor (TNF)-α and interleukin (IL)-6 mRNA levels were measured in proximal colon, adipose tissue and liver using RT-qPCR. Adipose macrophage infiltration (F4/80^+^) was assessed using immunohistochemical staining. HFD mice had a higher insulin/glucose ratio (*P* = 0.020) and serum levels of serum amyloid A3 (131%; *P* = 0.008) but reduced circulating adiponectin (64%; *P* = 0.011). In proximal colon of HFD mice compared to mice fed the control diet, transepithelial resistance and mRNA expression of zona occludens 1 were reduced by 38% (*P*<0.001) and 40% (*P* = 0.025) respectively and TNF-α mRNA level was 6.6-fold higher (*P* = 0.037). HFD reduced *Lactobacillus* (75%; *P*<0.001) but increased *Oscillibacter* (279%; *P* = 0.004) in fecal microbiota. Correlations were found between abundances of *Lactobacillus* (r = 0.52; *P* = 0.013) and *Oscillibacter* (r = −0.55; *P* = 0.007) with transepithelial resistance of the proximal colon. HFD increased macrophage infiltration (58%; *P* = 0.020), TNF-α (2.5-fold, *P*<0.001) and IL-6 mRNA levels (2.5-fold; *P* = 0.008) in mesenteric fat. Increased macrophage infiltration in epididymal fat was also observed with HFD feeding (71%; *P* = 0.006) but neither TNF-α nor IL-6 was altered. Perirenal and subcutaneous adipose tissue showed no signs of inflammation in HFD mice. The current results implicate gut dysfunction, and attendant inflammation of contiguous adipose, as salient features of the metabolic dysregulation of diet-induced obesity.

## Introduction

Visceral adiposity is strongly related to metabolic dysfunction including insulin resistance and systemic inflammation [1]–[3]. The deleterious metabolic effect of visceral fat is a consequence, in large part, of increased production of pro-inflammatory cytokines [4]. Adipose-derived immune factors primarily originate from cells in the stromal-vascular fraction of fat depots [5], [6]. Specifically, macrophage infiltration has been identified as a major determinant of the metabolic effect of adipose tissue. The abundance of macrophages in visceral fat was negatively correlated with insulin sensitivity of obese individuals, whereas no such relationship was observed with subcutaneous fat [7]. The primary driver of the macrophage infiltration and pro-inflammatory profile of visceral fat, however, remains largely unknown.

Evidence is now persuasive that the gut may play an important role in inducing adipose inflammation and metabolic disorders. In patients with Crohn's disease, the inflamed gut is associated with an expanded mesenteric fat depot characterized by an increased infiltration of immune cells [8]. A causal relationship between gut inflammation and mesenteric fat dysfunction (notably a relative expansion in size and an increase in expression of macrophages and immune factors) has been demonstrated in animal models of experimental colitis [9], [10]. Impaired gut barrier function, along with gut inflammation, have also been observed in both genetic [11] and dietary [12], [13] models of obesity.

Finally, there is an emerging body of literature demonstrating an association between altered gut microbiota profile and obesity in humans (see [14] and [15] for a recent review) which, from pioneering work on rodents [12], [16], [17], appears to be causative. The cross-linkage between gut microbiota, metabolic dysfunction and the immune system is best illustrated in Toll-like receptor-5 knockout mice which develop spontaneous colitis, insulin resistance, hyperlipidemia and increased visceral fat deposition [18]. Importantly, all of these deleterious effects can be ameliorated by antibiotic administration, implicating gut microbiota change as causative and suggesting a feedback loop from the innate immune system to gut inflammation and microbiota profile.

Taken together the case can be made, see [19], that obesogenic diets induce gut dysfunction, which may lead to the stimulation of contiguous fat by an adverse microbial load and subsequently results in visceral fat inflammation and systemic metabolic dysregulation.

The aim of the present study, therefore, was to characterize the relationship between gut health, visceral fat dysfunction and metabolic dysregulation in diet-induced obese mice. We showed that a prolonged high saturated fat feeding induced inflammation and impaired barrier function in the gut which associated with specific alterations in microbiota profile. This, together with a distinct pro-inflammatory nature of the mesenteric fat contiguous with the gut but not of depots more remote, implicates the gut in the genesis of visceral adipose inflammation and systemic metabolic dysfunction.

## Methods

### Ethics statement

All procedures were approved by the University of Sydney Animal Ethics Committee (approval number K00/2-2010/3/5225).

### Animals

Female C57BL/6J mice were obtained from the Australian Animal Resources Centre (Perth, Australia), group-housed (2–4 mice per cage) and kept with regulated temperature (18–22°C) and humidity (∼50%) with a 12 h light/dark cycle. Starting at 16 weeks of age, the mice were fed either a control (10% kcal from fat) or a high saturated fat diet (HFD; 60% kcal from fat, of which 34% was saturated fat) *ad libitum* with free access to water for 8 or 12 weeks. For each mouse fresh stools (1 pellet per day) were collected on 3 consecutive days in the week before termination for gut microbiota profiling. At termination mice were anesthetized after a 4 h fast. Blood was taken by heart puncture and serum was stored at −80°C. After flushing with ice-cold bicarbonate buffer [20], segments of proximal colon (immediately below the cecum) were taken for assessment of gut permeability and gene expression (see below). Mesenteric, epididymal, perirenal and subcutaneous fat samples were taken for immunohistochemical staining and gene expression analysis, as was liver for the latter measurements.

### Biochemical analyses

Blood glucose was measured using a glucometer (Accu-Chek Performa, Roche, Mannheim, Germany). Serum concentrations of insulin, adiponectin and serum amyloid A3 were measured using assays from Millipore (Billerica, MA, USA). Liver was homogenized in isopropanol. Liver triglycerides and serum concentrations of alanine transaminase and triglycerides were measured using kits from Horiba ABX (Montpellier, France) and the assays were performed on the ABX Pentra 400 (Horiba ABX). Serum concentrations of interleukin (IL)-1β, IL-10, monocyte chemotactic protein (MCP)-1, tumor necrosis factor (TNF)-α, IL-6, IL-12p40 and plasminogen activator inhibitor (PAI)-1 were determined using commercially available cytokine kits and the xMAP® technology (Luminex Corporation, Austin, TX, USA). The data were analyzed with a four- or five-parametric curve fitting using the Bio-Plex Manager Software (version 5.0, Life Science Research, Hercules, CA, USA).

### Assessment of gut permeability

Gut permeability was measured as described by Wang et al [21] with minor modifications. The segments of proximal colon for gut permeability measurement were opened along the mesenteric border and mounted in the Ussing chamber with an aperture of 0.3 cm^2^. The chamber was connected to a VCC MC6 amplifier, controlled and monitored using the Acquire & Analyze software (V2.3.177 Physiologic Instruments, San Diego, CA, USA). Experiments were carried out under current-clamp (open-circuit) conditions as described previously [22]. Segments of colon were incubated in oxygenated (95% O_2_; 5% CO_2_) bicarbonate buffer at 37°C [20]. Tissue was equilibrated for 15 min, thereafter a 3 µA current pulse was applied across the intestinal wall every 6 sec for 30 min. The transepithelial potential was measured and recorded by the Acquire & Analyze software, and the change in potential induced by the current pulse was used to calculate transepithelial resistance according to Ohm's Law.

### Quantitative real-time PCR

RNA was extracted from homogenized tissue using TRI reagent (Sigma-Aldrich, St Louis, MO, USA) and isolated by phenol/chloroform extraction. Total RNA (200 ng) was reverse transcribed to cDNA using BioScript™ (Bioline, London, UK) and random hexamers (Applied Biosystems, Carlsbad, CA, USA). Primer sequences for sterol regulatory element binding protein (SREBP)-1c [23], peroxisome proliferator-activated receptor (PPAR)-γ [23], proglucagon [24], zona occludens (ZO)-1 [25], occludin [26] and IL-10 [27] have been published elsewhere. Primers for SCD1, TNF-α and IL-6 were purchased from Qiagen (Valencia, CA, USA). mRNA expression was quantified by RT-qPCR using the Rotorgene™ 3000 Real Time Thermal Cycler (Qiagen). Fluorescence data were analyzed using the Rotor-Gene 6 software (version 6.0, Qiagen). mRNA expression was normalized to RPL-19 [24], UBC [28] and 18S rRNA [29] for colon, adipose tissue and liver respectively and reported as arbitrary units.

### Immunohistochemical staining

Fat samples were fixed in formalin overnight and embedded in paraffin. Sections of 10 µm thickness were treated with 10 µg/ml proteinase K (Sigma-Aldrich) at room temperature for antigen retrieval. Endogenous peroxidase and biotin signals were minimized using 0.3% (v/v) hydrogen peroxide and avidin/biotin block (Dako, Glostrup, Denmark) respectively. After incubating with 10% (v/v) normal horse serum to reduce non-specific staining, sections were incubated with purified anti-mouse F4/80 antibody (BioLegend, San Diego, CA, USA) for 1 h and subsequently with biotinylated rabbit anti-rat IgG antibody (Vector Laboratories, Burlingame, CA, USA) for 30 min at room temperature. The expression of F4/80 was detected using the VECTASTAIN^®^ ABC Kit (Vector Laboratories) and the Liquid DAB+ Substrate Chromogen System (Dako). All sections were counterstained with hematoxylin. F4/80^+^ cells were quantified by counting the number of adipocytes and F4/80^+^ cells in 10 random areas at ×40 magnification and the abundance of F4/80^+^ cells was normalized to 100 adipocytes [30]. Adipocyte size was determined by averaging the cross-sectional area of 5 adipocytes of each of the 10 selected ×40 magnification field using ImageJ (version 1.44p, National Institutes of Health, MD, USA).

### Gut microbiota profiling

For each mouse three stool pellets (one per day) were pooled and homogenized in TE buffer (pH 7.5). DNA was extracted from 0.5 ml of homogenate using a bead beating method as described previously [31] and stored at −20°C. Metagenomic DNA (>500 ng from each mouse) was sent to Research and Testing Laboratories (Lubbock, USA) for bacterial tag-encoded FLX amplicon pyrosequencing (bTEFAP) of the V6–V9 region of the 16S rDNA. Modified versions of primers F939 (5′TTGACGGGGGCCCGCAC3′) and R1492 (5′ATTAGATACCCNGGTAG3′) incorporating domains for 454 sequencing and unique identification tags were used to allow the identification of sequences from individual sample. Amplicons were sequenced on a GS Pico Titer Plate using the Roche 454 FLX instrument with corresponding FLX titanium reagents (Roche Applied Science, Indianapolis, IN, USA).

bTEFAP raw data were processed using Mothur [32] unless stated otherwise. Parameters used to filter sequences for analysis were based upon published studies [33], [34]. Briefly, sequences from the raw 454 data were quality-filtered to exclude those with an average Phred score <30. Remaining sequence reads were binned into individual sample sets by the unique tags with sequences >200 nucleotides in length (excluding primers and tags) retained. The sequences were then screened to remove chimeras using chimera slayer within Mothur to give a final set of quality-filtered sequence reads for each sample. Taxonomic classifications were assigned using the naïve Bayesian algorithm with the Ribosomal Database Project (RDP) Training Set 6 as a reference database. No new data were generated from DNA sequencing. Nomenclature of all bacterial identification was based on Bergey's taxonomic outline. Within each sample the number of sequences for each bacterial taxonomic assignment is expressed as a percentage of the total reads retained after quality-filtering (relative abundance). To quantify the coverage and sampling effort of bTEFAP, RDP pyrosequencing pipeline was used to generate rarefaction curves of the sequences.

### Statistical analysis

Data are expressed as means ± SEM. Student's *t*-test, one-way or two-way ANOVA were used to compare data from different treatment groups. Corrections of *p*-values for multiple testing were performed using Bonferroni post hoc tests. Relationships between selected outcome variables were examined by Pearson's correlation. Statistical analyses were performed using the GraphPad Prism Program (version 5.01, GraphPad Software Inc., San Diego, CA, USA). Significance was accepted at *P*<0.05.

## Results

### High saturated fat diet induced weight gain, systemic insulin resistance and inflammation

**Figure 1:**
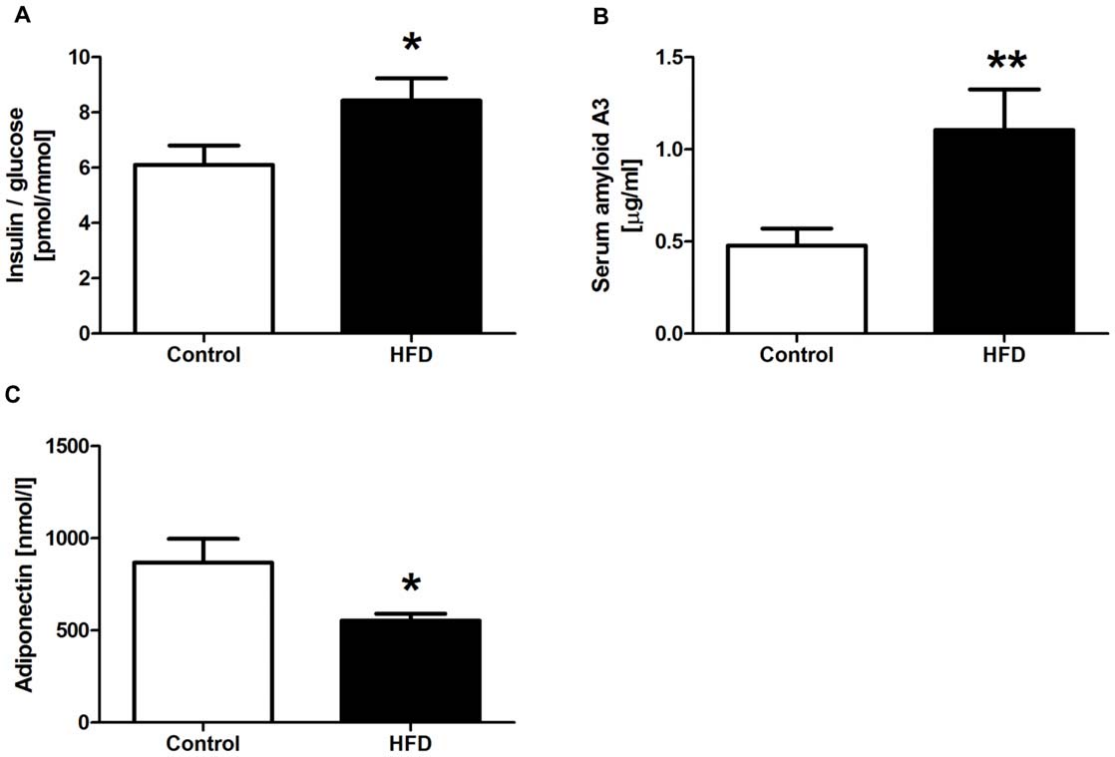
Serum biochemical analyses. Insulin/glucose ratio (A), concentrations of serum amyloid A3 (B) and adiponectin (C) in control and high saturated fat diet (HFD) fed mice (n = 15–16 per group). Data are shown as mean ± SEM. **P<0.01 and *P<0.05 compared to control.

By the end of the 8- and 12-week feeding protocols, the body weights of HFD mice were 22% and 36% higher respectively than animals fed the control diet (27.3±0.7 g vs 22.4±0.5 g for controls; *P*<0.001 at 8 weeks and 30.1±1.1 g vs 22.2±0.4 g for controls; *P*<0.001 at 12 weeks). Eight and 12 week results were not different for all other outcome variables and were therefore combined for analyses. HFD mice had a higher insulin/glucose ratio (1.4-fold; *P* = 0.020; insulin 91.1±8.8 pmol/l vs 56.8±6.8 pmol/l for controls; glucose 10.9±0.3 mmol/l vs 9.4±0.5 mmol/l for controls) as a marker of insulin resistance (Figure 1A). Circulating levels of the acute phase inflammatory marker serum amyloid A3 were 2.3-fold (*P* = 0.008; Figure 1B) higher in HFD mice, but in contrast, those of adiponectin were decreased by 36% (*P* = 0.011; Figure 1C). Serum concentrations of triglycerides, IL-1β, IL-10, MCP-1, TNF-α, IL-6, IL-12p40 and PAI-1 were not different between the diet groups (data not shown).

### High saturated fat diet increased gut permeability and inflammation

**Figure 2:**
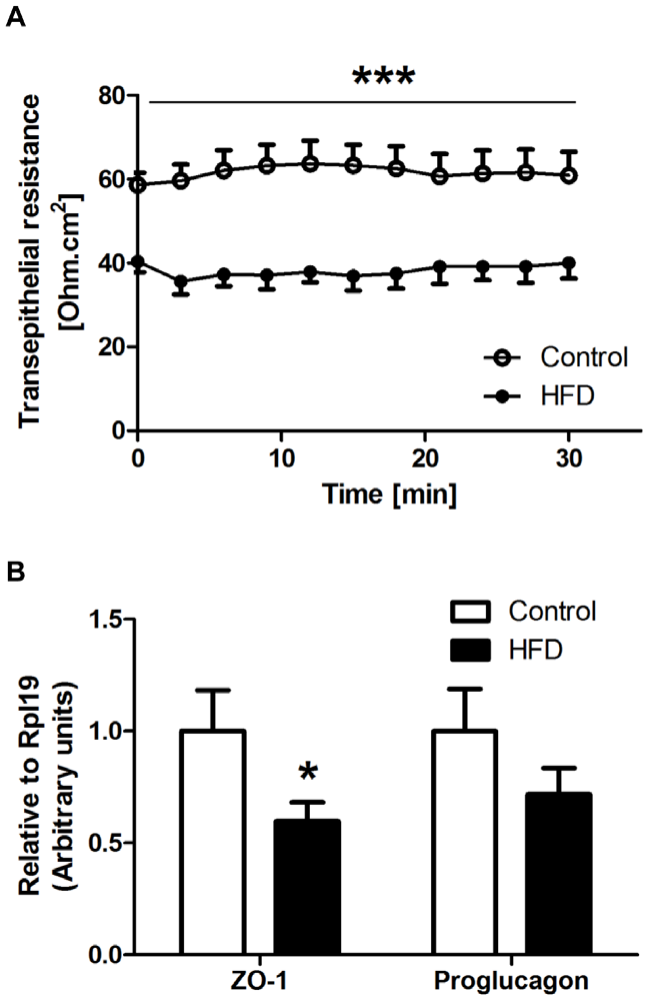
Effect of high saturated fat diet (HFD) on barrier function of proximal colon. A: Transepithelial resistance of mouse proximal colon was determined in the Ussing chamber by measuring the change in potential difference in response to 3 µA current generated across the tissue segment. B: mRNA levels of zona occludens (ZO)-1 and proglucagon were measured using RT-qPCR. Gene expression was normalized to RPL-19. Open circles/bars = control; Closed circles/bars = HFD (n = 9–16 per group). Data are shown as mean ± SEM. ***P<0.001 and *P<0.05 compared to control.

**Figure 3:**
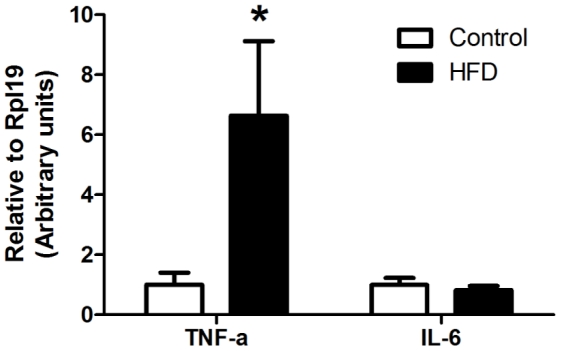
Effect of high saturated fat diet (HFD) on markers of inflammation in proximal colon. mRNA expression of indicated genes was measured by RT-qPCR as described in Figure 2. Open bars = control; Closed bars = HFD (n = 11–16 per group). Data are shown as mean ± SEM. *P<0.05 compared to control.

In the proximal colon, transepithelial resistance of HFD mice was reduced by 38% (37.4±2.6 Ohm.cm^2^ vs 60.8±4.5 Ohm.cm^2^ for controls; *P*<0.001; Figure 2A) as compared to controls. mRNA expression of ZO-1 and proglucagon were also reduced by 40% (*P* = 0.025) and 28% (*P* = 0.104) respectively in HFD mice (Figure 2B), whereas the mRNA levels of occludin were not different between the diet groups (data not shown). The colon tissue of the HFD treatment group had increased mRNA expression of TNF-α by 6.6-fold (*P* = 0.037) but that of IL-6 was unchanged (Figure 3).

### High saturated fat diet altered gut microbiota profile

**Figure 4:**
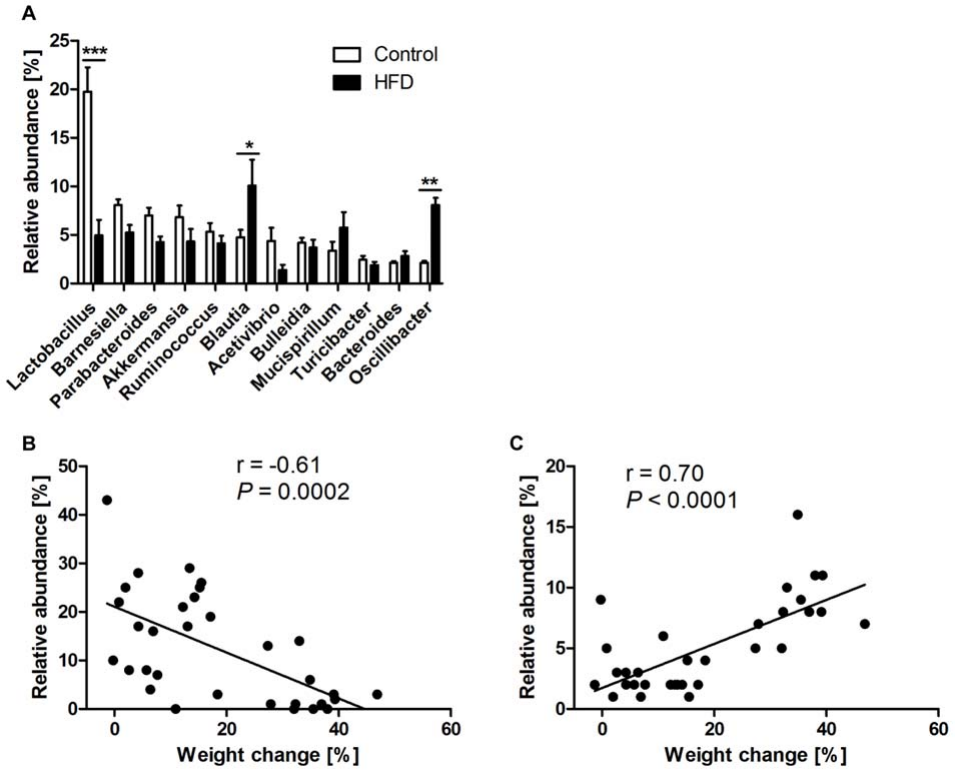
Gut microbiota composition in control and high saturated fat diet (HFD) fed mice. A: Relative abundance of main genera in fecal samples from control (open bars) and HFD (closed bars) mice (n = 16 per group). Data are shown as mean ± SEM. ***P<0.001, ** *P*<0.01 and *P<0.05 compared to control. Correlations between the abundance of *Lactobacillus* (B) and *Oscillibacter* (C) and weight changes. Gut microbiota profile was determined by metagenomic pyrosequencing from bacterial lineages in fecal samples.

After quality-filtering a total of 246,694 sequences were obtained from 16 control and 16 HFD mice with a mean of 7,709 sequences per mouse (range 1,758–15,005; Table S1). The average number of reads for the control diet sample series was higher than that from HFD, however rarefaction analysis showed that taxonomic richness had plateaued in all samples at higher taxon level. Rarefaction curves at 95% sequence identity, considered to approximate genus level, showed that the rate of sampling was comparable across all samples and demonstrated sampling depth at this taxonomic resolution was comparable to previously reported studies (Figure S1) [35]–[37]. At the phylum level, HFD mice had more Firmicutes (73±1.5% vs 68±2.3% for controls; *P* = 0.041) and fewer Bacteroidetes (15±1.7% vs 19±1.3% for controls; *P* = 0.026) and thus a significantly higher Firmicutes: Bacteroidetes ratio (*P* = 0.019). HFD also induced significant shifts in fecal microbiota composition at the genus level of taxonomic resolution. Within members of the Firmicutes, notably there was a 75% decrease in *Lactobacillus* (*P*<0.001) and a 279% increase in *Oscillibacter* (*P* = 0.004) as compared to controls and these changes in abundance were closely associated with weight gain (Figure 4).

**Figure 5:**
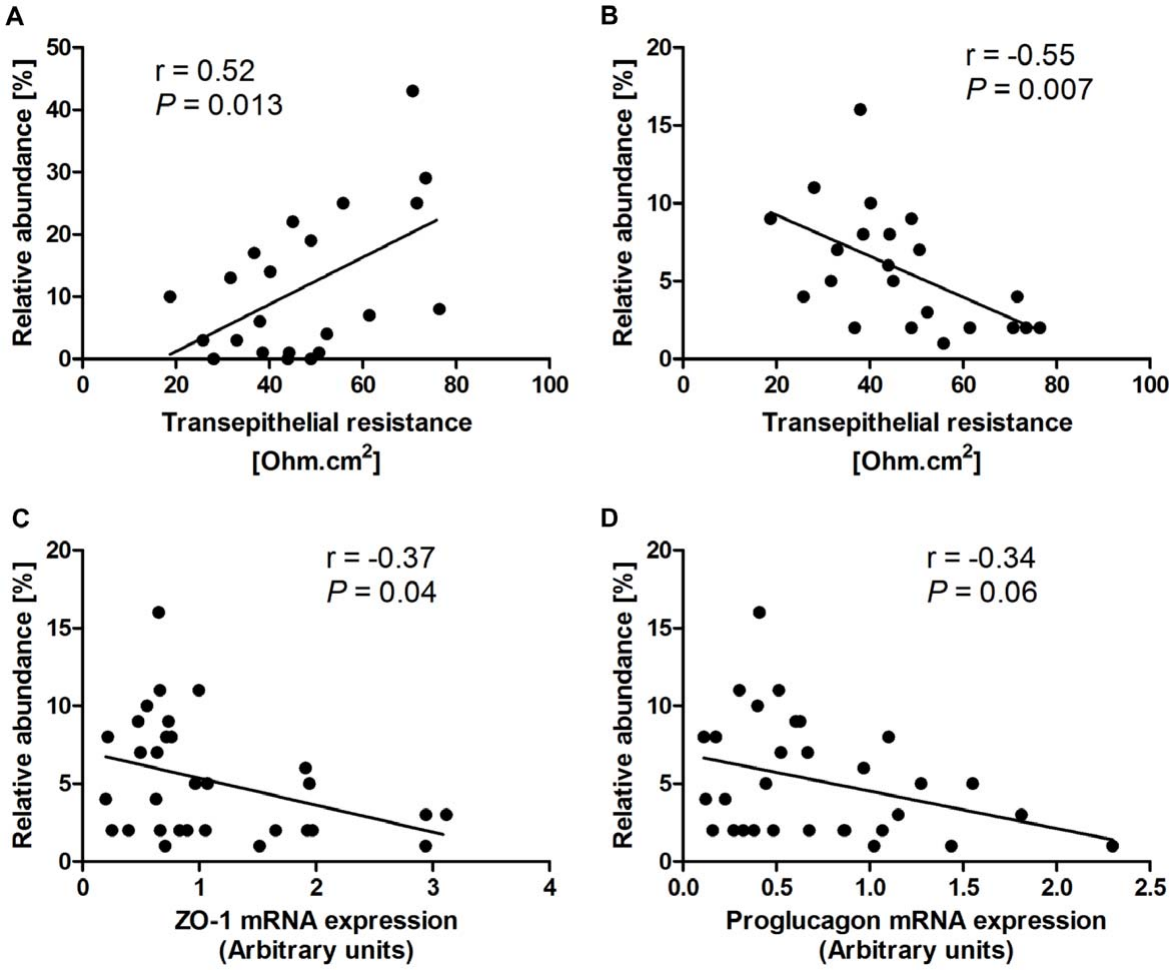
Correlations between *Lactobacillus* (A) and *Oscillibacter* (B–D) abundances and permeability parameters in proximal colon. Gut microbiota profile was determined as described in Figure 4 (n = 22–31). Transepithelial resistance and mRNA expression of zona occludens (ZO)-1 and proglucagon in proximal colon were measured as described in Figure 3.

Transepithelial resistance of the proximal colon was positively correlated with the abundance of *Lactobacillus* (r = 0.52; *P* = 0.013; Figure 5A) but negatively correlated with that of *Oscillibacter* (r = −0.55; *P* = 0.007; Figure 5B). Notably, increased *Oscillibacter* abundance was also associated with a reduction in the mRNA expression of ZO-1 (r = −0.37; *P* = 0.039; Figure 5C) and, although not statistically significant, a similar trend was observed with proglucagon (r = −0.34; *P* = 0.061; Figure 5D).

### High saturated fat diet induced depot-specific inflammation and altered morphology in adipose tissue

**Figure 6:**
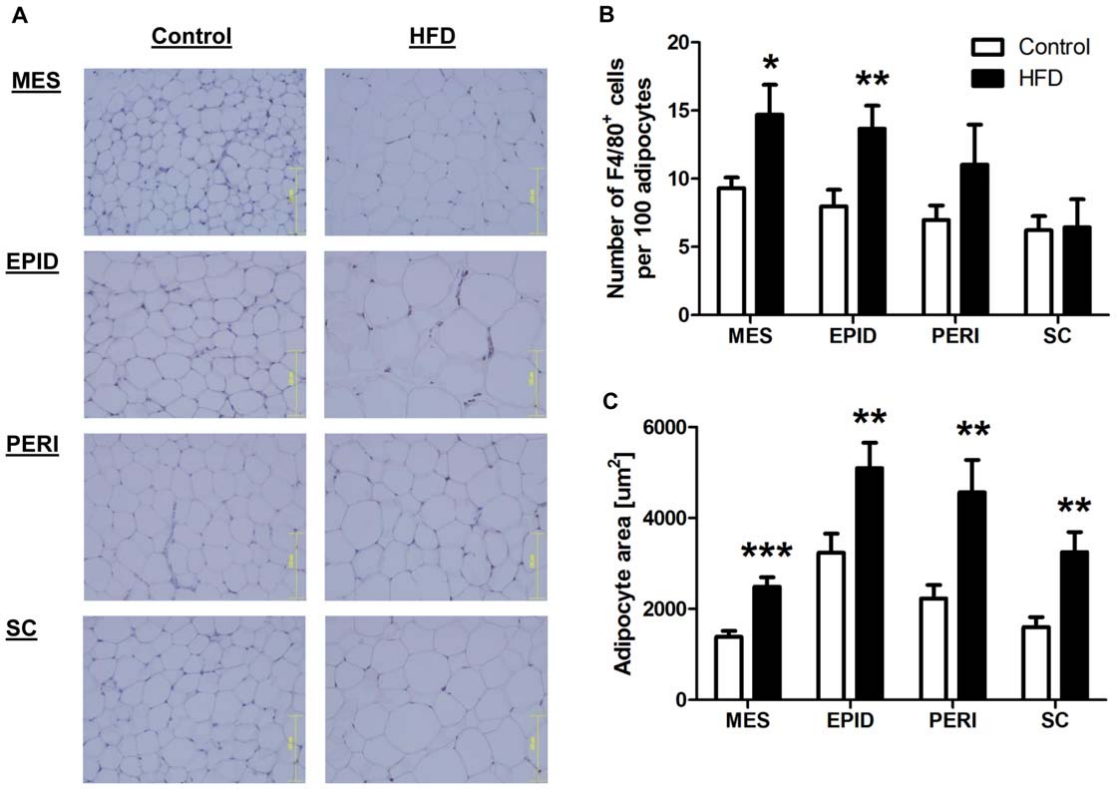
Effect of high saturated fat diet (HFD) on adipose macrophage infiltration and adipocyte size. A: Representative immunohistochemical staining of mesenteric (MES), epididymal (EPID), perirenal (PERI) and subcutaneous (SC) fat. Quantification of macrophage (F4/80^+^) infiltration (B) and adipocyte size (C). Tissues were fixed in formalin and embedded in paraffin. Sections were stained for F4/80 and counterstained with hematoxylin. Open bars = control; Closed bars = HFD (n = 4–10 per group). Data are shown as mean ± SEM. ***P<0.001, **P<0.01 and *P<0.05 compared to control.

Representative immunohistochemical staining of the macrophage marker F4/80 in adipose tissue of control and HFD mice is shown in Figure 6A. The abundance of macrophages (F4/80^+^ cells) was similar across all fat depots in the control mice. HFD increased macrophage infiltration in mesenteric (58%; *P* = 0.020) and epididymal (71%; *P* = 0.006) fat but was without effect in perirenal and subcutaneous fat (Figure 6B). HFD increased adipocyte size in mesenteric (80%; *P*<0.001), epididymal (57%; *P* = 0.008), perirenal (104%; *P* = 0.007) and subcutaneous (103%; *P* = 0.008) fat (Figure 6C). In both diet groups, mesenteric fat was the depot with the smallest adipocytes (Figure 6C).

**Figure 7:**
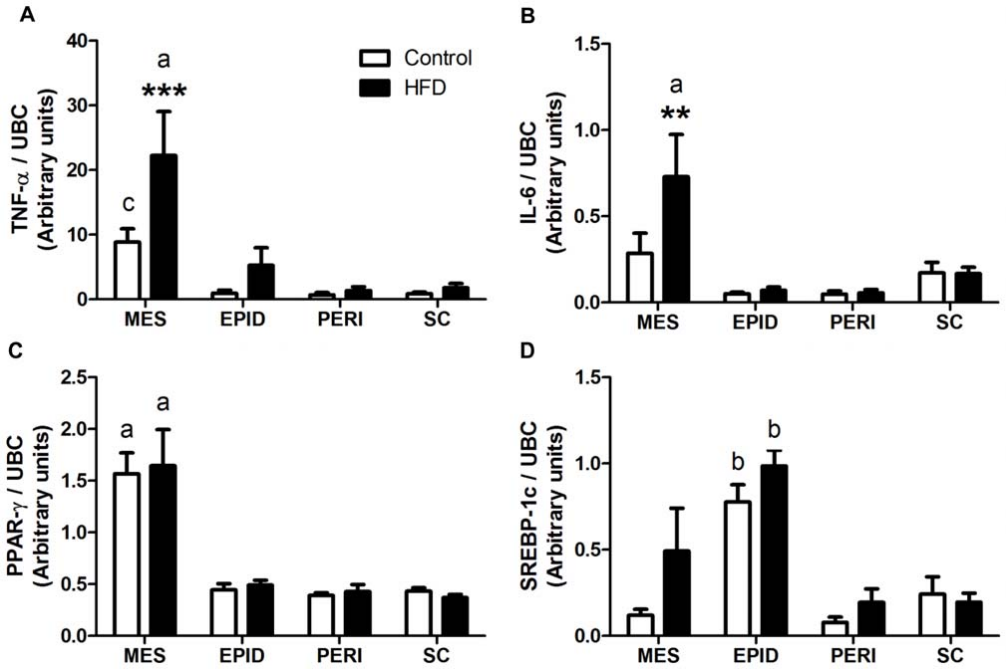
mRNA expression of TNF-α (A), IL-6 (B), PPAR-γ (C) and SREBP-1c (D) in adipose tissue. mRNA levels in control and high saturated fat diet (HFD) fed mice were measured by RT-qPCR. Gene expression was normalized to UBC. Open bars = control; Closed bars = HFD (n = 12–15 per group). Data are shown as mean ± SEM. ***P<0.001 and **P<0.01 compared to control. a, b, c: *P*<0.001, *P*<0.01 and *P*<0.05 respectively compared to other depots in the same diet group.

Compared to controls, HFD induced a 2.5-fold increase in the mRNA levels of both TNF-α (*P*<0.001; Figure 7A) and IL-6 (*P* = 0.008; Figure 7B) in the mesenteric fat. This depot had by far the highest expression of TNF-α and IL-6 in mice fed the control diet, with expression levels being very low, and unaffected by HFD, in epididymal, perirenal and subcutaneous depots (Figures 7A and B). HFD had no effect on the mRNA expression of SREBP-1c and PPAR-γ in adipose tissue. In both diet groups, the highest expression of PPAR-γ (*P*<0.001; Figure 7C) and SREBP-1c (*P* = 0.003; Figure 7D) were found in mesenteric and epididymal fat respectively.

### High saturated fat diet led to a pro-inflammatory and lipogenic liver

**Figure 8:**
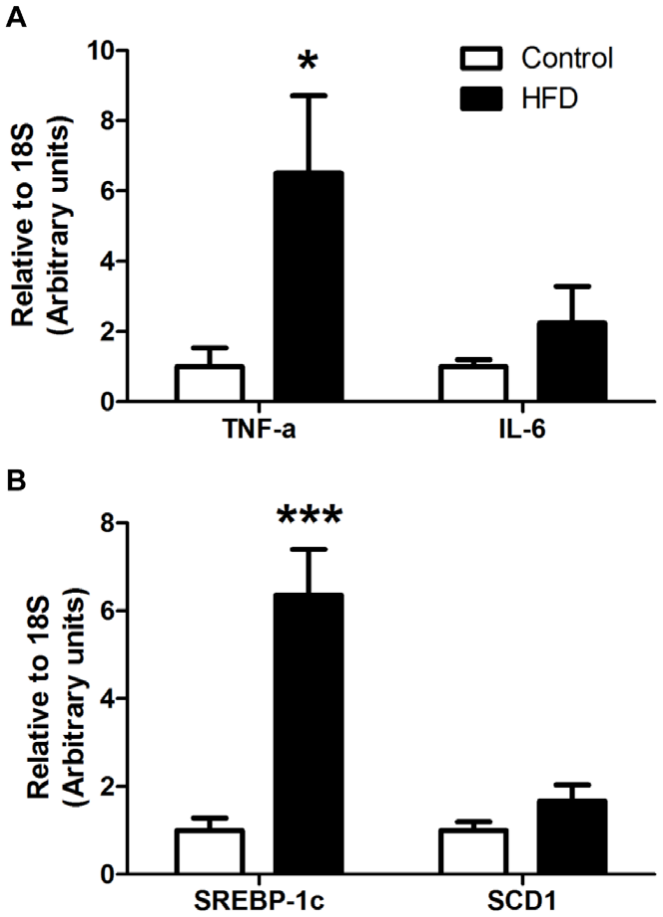
mRNA expression of genes involved in inflammation (A) and lipogenesis (B) in liver. mRNA levels of TNF-α, IL-6, SREBP-1c and SCD1 in control and high saturated fat diet (HFD) fed mice were measured using RT-qPCR. Gene expression was normalized to 18S. Open bars = control; Closed bars = HFD (n = 14–16 per group). Data are shown as mean ± SEM. ***P<0.001 and *P<0.05 compared to control.

As shown in Figure 8A, HFD-fed mice tended to have higher expression of pro-inflammatory factors in the liver, as evidenced by the increased TNF-α (6.5-fold; *P* = 0.018) and IL-6 (2.2-fold; *P* = 0.137) mRNA expression. Hepatic SREBP-1c (6.4-fold; *P*<0.001) and SCD1 (1.7-fold; *P* = 0.069) mRNA levels were also trended to be elevated in HFD mice (Figure 8B).

## Discussion

The present study aimed to determine the effect of a high, predominantly saturated, fat diet (HFD) on gut health and its relationship with metabolic dysfunction. HFD mice exhibited systemic inflammation, whole-body insulin resistance and liver inflammation. We showed that HFD increased gut permeability and altered gut microbiota profile. Importantly, we demonstrated a relationship between gut barrier function and the abundance of specific genera of the microbial community. Together with the evidence of inflammation in the proximal colon and the distinct pro-inflammatory nature of the contiguous mesenteric, but not other fat depots, the present results highlight gut dysfunction as a salient feature of diet-induced metabolic dysregulation.

Our data suggest that HFD initiates metabolic changes that impaired gut barrier function, as evidenced both by the decrease in transepithelial resistance and mRNA expression of ZO-1 in the proximal colon. Similar outcomes for other obesogenic diets on gut barrier function have also been reported. Cani et al [12] showed an increase in whole-gut permeability in HFD mice, an effect associated with a reduction in mRNA expression of tight-junction proteins including ZO-1. Little is known, however, about the barrier integrity of functionally distinct regions of the intestine in diet-induced models of obesity. The present study focused directly on the proximal colon, which is the part of the gut predicted to have the most intense microbial activity. Accordingly, loss of barrier integrity in the proximal colon has the potential to induce a disproportionate effect of gut microbiota on systemic metabolism.

The increased permeability of the proximal colon is postulated to participate in a feedback loop with inflammatory processes. In our study, and others using obesogenic diets, mice in the experimental groups had increased TNF-α mRNA expression [37] in the intestine. In an experimental model of colitis, the progressive increase in colonic permeability is associated with a corresponding reduction in ZO-1 protein expression [38]. There is also some evidence suggesting a direct effect of TNF-α on gut barrier integrity. Anti-TNF-α treatment improves gut barrier function in patients with Crohn's disease [39]. In Caco-2 cell cultures, TNF-α reduced transepithelial resistance and ZO-1 protein expression via a NFκB-dependent pathway [40]. The effect of TNF-α on NFκB activation also increased the expression and activity of myosin light chain kinase, which subsequently leads to disorganization of tight-junction proteins at the intestinal barrier [41]. It is worth noting that other cytokines upstream of NFκB, including IL-1β [42] and IFN-γ [43], also have been shown to decrease tight-junction function. The collective role of inflammatory pathways, rather than individual cytokines, therefore, appears to be critical in the regulation of gut barrier function.

The activity of the microbiota is a major determinant of gut health and a ‘poor’ microbiota composition has been linked to obesity and metabolic dysfunction [44]. The shift in the ratio of Firmicutes and Bacteroidetes by obesogenic diets has been frequently reported, but not universally so. Here we observed a small, but significant shift towards an increased Firmicutes: Bacteroidetes ratio in the HFD mice. The most startling microbiota changes were observed within the Firmicutes, with a very strong association between changes in relative abundance of *Lactobacillus* and *Oscillibacter* and the two diet groups. Members of the genus *Lactobacillus* have been intensively investigated for probiotic properties and several strains have been shown to ameliorate gut inflammation [45] and enhance barrier function [46] in experimental models of gut dysfunction. A novel finding here is the positive correlation between the abundance of the indigenous *Lactobacillus* community (i.e. not administered probiotic strains) and barrier function of the proximal colon. However we did not observe a significant correlation between *Lactobacillus* content and mRNA expression of tight-junction proteins. The effect of probiotic *Lactobacillus* strains on tight-junction integrity is not entirely clear and appears to be strain-specific [47], [48]. In humans, administration of *L. plantarum* promoted localization of ZO-1 and occludin in the tight-junctions without necessarily affecting their transcriptional levels [49]. It is worth noting that the beneficial effect of *Lactobacillus* on gut health may not be restricted to the maintenance of barrier integrity, as there is some evidence suggesting that certain strains may alter the expression of defensin, an anti-microbial peptide, which is important for mucosal protection [50], [51].

The present study identified *Oscillibacter*-like organisms as a potentially important gut microbe that mediates HFD-induced gut dysfunction. This group, including *Oscillibacter* and *Oscillospira*, are very poorly represented in culture collections but have been consistently detected in the microbial community of humans [52], [53]. Recent data suggest that the abundance of *Oscillibacter* is diet-responsive in obese individuals [54] but to date little is known about its physiological role. The negative correlation between the abundance of *Oscillibacter* and parameters of barrier function in the proximal colon is intriguing. There is some *in vitro* evidence suggesting that metabolites from other gut microbes may modify the abundance of certain strains of *Oscillibacter* [55]. It is possible that *Oscillibacter* directly regulates components involved in the maintenance of gut barrier integrity, or its relationship with gut permeability may be a secondary effect consequent upon alterations in the overall composition of the microbial community. Characterization of the metabolic effect of *Oscillibacter* as well as its interactions with other microbes would be critical to elucidate its role in diet-induced metabolic dysfunctions.

We identified mesenteric fat as a metabolically distinct visceral fat depot with the most prominent pro-inflammatory nature. Our data showed that mesenteric fat has the highest mRNA expression of PPAR-γ and the smallest adipocyte size. This is in contrast to epididymal fat which had the biggest adipocytes and the highest mRNA content of SREBP-1c, a transcription factor that promotes lipogenesis. HFD mice had increased expression of TNF-α and IL-6 and macrophage infiltration in mesenteric fat [13], [56] and the role of TNF-α in the systemic dysmetabolism may be particularly critical [57]. Similar trends of depot-specific differences in the expression of pro-inflammatory cytokines have been reported previously in animal models of high-fat feeding [58], [59].

A range of non-adipocytes cells are known to contribute strongly to the pro-inflammatory secretory profile of adipose tissue [6]. Interestingly, the HFD-induced increase in macrophage infiltration was similar in mesenteric and epididymal fat, and yet only the former had a distinctly elevated expression of pro-inflammatory cytokines. A limitation of our study is that we used only F4/80 as a macrophage marker, which did not allow us to distinguish sub-populations of macrophages. The increased transcriptional expression of pro-inflammatory factors in the mesenteric fat may be a consequence of the preferential infiltration of macrophages with a pro-inflammatory phenotype [60]. Equally, the presence of lymph nodes in mesenteric [61], but not epididymal, fat implies that immune cells other than macrophages, e.g. B-cells, T-cells and Natural Killer cells, are likely to contribute to the secretory function of the mesenteric depot.

The increased macrophage infiltration and TNF-α expression in mesenteric fat of our HFD mice is consistent with results in rats with induced gut inflammation [9] and is likely to be a consequence of the ‘leakage’ of gut luminal content. Cenac et al [62] demonstrated the sequential occurrences of gut inflammation, impaired barrier function and bacterial translocation to the mesenteric lymph nodes in an experimental model of colitis. Similar metabolic sequelae of gut inflammation in obesity are yet to be reported. However, the recent report of bacterial translocation into the mesenteric fat of HFD mice [63] and of elevated circulating endotoxin levels in animal models of diet-induced obesity [12] and in obese and type 2 diabetic patients related to insulin resistance [64], [65] are consistent with our hypothesized role of the gut in metabolic dysfunction [19].

Mechanistic studies will be an important extension of the current work. We, and others, have shown that an obesogenic diet can induce gut dysfunction involving multiple feedbacks with immune and metabolic regulation. It remains unclear whether HFD-induced gut dysfunction is a specific effect of fatty acid subtype (e.g. saturated fatty acids are known to be pro-inflammatory) or is merely a consequence of energy overload. Elucidating the sequence of factors that initiates gut dysfunction will be important for the maintenance of optimal metabolic health. From the disease treatment perspective, the interactions and feedback mechanisms between the innate immune system and the microbiota profile and their effect on metabolic outcomes [18] imply that a combination of pharmaceutical interventions of immunomodulators (e.g. pro-resolving mediators that are critical in maintaining immunological homeostasis of mucosal surface [66]) and manipulation of microbiota (e.g. dietary supplementation of prebiotics, probiotics and resistant starches) may be novel gut-targeted approaches to ameliorate metabolic dysfunctions.

In summary, the current findings build on a literature now becoming compelling that the gut is a central player in the aetiology of diet-induced metabolic diseases.

## Supporting Information

Figure S1 **Rarefaction curves at 90% (A) and 95% (B) sequence identity depicting the number of operational taxonomic units (OTUs) against sampling effort (n = 16 per group).** Curves were generated based on the pool of 246,694 high quality sequences. The number of OTUs present in individual samples was sorted by control (C) and high-fat diet (F).
(TIF)

Table S1 **Number of sequences in each sample before and after quality trimming.**
(DOC)
