# Supplementary figures and images for: Increased Gut Permeability and Microbiota Change Associate with Mesenteric Fat Inflammation and Metabolic Dysfunction in Diet-Induced Obese Mice
Source: PLoS One. 2012 Mar 23;7(3):e34233. doi: 10.1371/journal.pone.0034233 (PMC3311621; doi:10.1371/journal.pone.0034233)

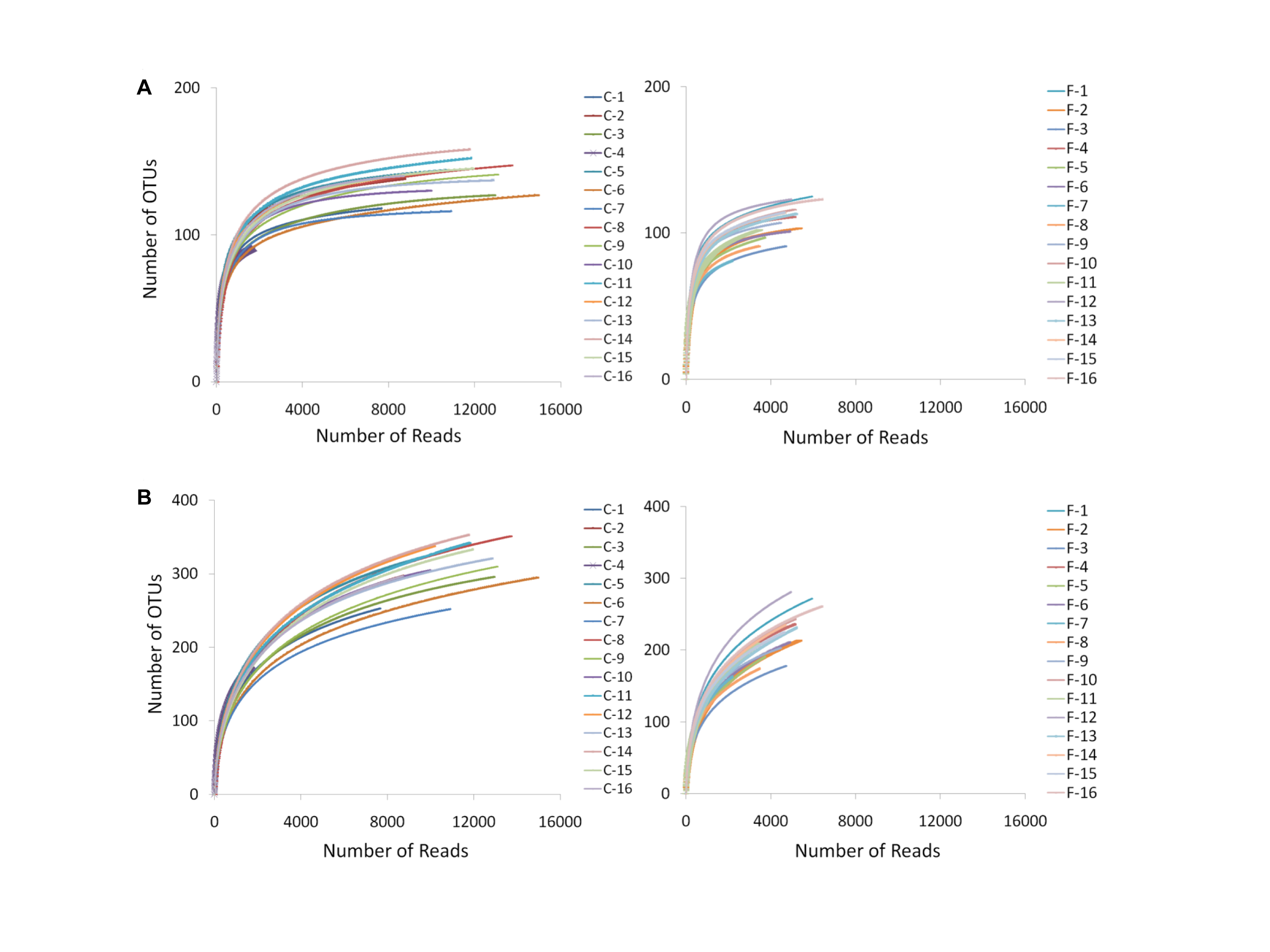

Supplement: Figure S1 — Rarefaction curves at 90% (A) and 95% (B) sequence identity depicting the number of operational taxonomic units (OTUs) against sampling effort (n = 16 per group). Curves were generated based on the pool of 246,694 high quality sequences. The number of OTUs present in individual samples was sorted by control (C) and high-fat diet (F). (TIF) [file pone.0034233.s001.tif]
