# Supplementary material for: Increased Gut Permeability and Microbiota Change Associate with Mesenteric Fat Inflammation and Metabolic Dysfunction in Diet-Induced Obese Mice
Source: PLoS One. 2012 Mar 23;7(3):e34233. doi: 10.1371/journal.pone.0034233 (PMC3311621; doi:10.1371/journal.pone.0034233)
Supplement: Table S1 — Number of sequences in each sample before and after quality trimming. (DOC) [file pone.0034233.s002.doc]

| **Mouse** | **Sequences obtained from bTEFAP** | **Sequences with > 200 nucleotides and an average Phred score > 30** | **Non chimeric sequences with bacterial classification** | **Sequences removed** |
| --- | --- | --- | --- | --- |
|
| C-1 | 11,035 | 8,131 | 7,688 | 30.33% |
| C-2 | 12,742 | 9,558 | 8,771 | 31.16% |
| C-3 | 19,289 | 14,092 | 12,963 | 32.80% |
| C-4 | 4,526 | 2,453 | 1,758 | 61.16% |
| C-5 | 16,647 | 12,410 | 10,819 | 35.01% |
| C-6 | 21,394 | 16,231 | 15,005 | 29.86% |
| C-7 | 16,537 | 12,136 | 10,929 | 33.91% |
| C-8 | 20,227 | 15,012 | 13,600 | 32.76% |
| C-9 | 19,658 | 14,920 | 13,110 | 33.31% |
| C-10 | 14,585 | 11,213 | 10,001 | 31.43% |
| C-11 | 17,336 | 13,082 | 11,840 | 31.70% |
| C-12 | 15,044 | 11,760 | 10,209 | 32.14% |
| C-13 | 19,024 | 14,324 | 12,887 | 32.26% |
| C-14 | 16,969 | 12,893 | 11,789 | 30.53% |
| C-15 | 18,404 | 13,303 | 11,969 | 34.97% |
| C-16 | 12,838 | 10,029 | 8,723 | 32.05% |
| F-1 | 10,648 | 5,448 | 5,955 | 44.07% |
| F-2 | 9,938 | 5,194 | 5,353 | 46.14% |
| F-3 | 8,901 | 4,445 | 4,738 | 46.77% |
| F-4 | 9,401 | 4,523 | 5,164 | 45.07% |
| F-5 | 6,428 | 3,276 | 3,742 | 41.79% |
| F-6 | 8,669 | 4,715 | 4,923 | 43.21% |
| F-7 | 8,423 | 4,473 | 2,201 | 73.87% |
| F-8 | 6,445 | 3,295 | 3,481 | 45.99% |
| F-9 | 8,853 | 3,908 | 4,498 | 49.19% |
| F-10 | 9,548 | 4,484 | 5,081 | 46.78% |
| F-11 | 6,532 | 3,427 | 3,499 | 46.43% |
| F-12 | 10,491 | 4,728 | 4,962 | 52.70% |
| F-13 | 9,167 | 4,617 | 5,233 | 42.91% |
| F-14 | 8,701 | 4,075 | 4,698 | 46.01% |
| F-15 | 8,861 | 3,992 | 4,662 | 47.39% |
| F-16 | 12,167 | 6,236 | 6,443 | 47.05% |
| Total | 399,428 | 262,383 | 246,694 | 38.24% |
